# Analysis of histology and long noncoding RNAs involved in the rabbit hair follicle density using RNA sequencing

**DOI:** 10.1186/s12864-021-07398-4

**Published:** 2021-01-28

**Authors:** Haisheng Ding, Huiling Zhao, Xiaowei Zhao, Yunxia Qi, Xiaofei Wang, Dongwei Huang

**Affiliations:** Anhui Key Laboratory of Livestock and Poultry Product Safety Engineering, Institute of Animal Husbandry and Veterinary Medicine, Anhui Academy of Agricultural Sciences, Hefei, 230031 Anhui People’s Republic of China

**Keywords:** Skin, Hair follicle density, Wool production, Histological analysis, LncRNA expression, RNA sequencing, Angora rabbit

## Abstract

**Background:**

Hair follicle density influences wool fibre production, which is one of the most important traits of the Wan Strain Angora rabbit. However, molecular mechanisms regulating hair follicle density have remained elusive.

**Results:**

In this study, hair follicle density at different body sites of Wan Strain Angora rabbits with high and low wool production (HWP and LWP) was investigated by histological analysis. Haematoxylin-eosin staining showed a higher hair follicle density in the skin of the HWP rabbits. The long noncoding RNA (lncRNA) profile was investigated by RNA sequencing, and 50 and 38 differentially expressed (DE) lncRNAs and genes, respectively, were screened between the HWP and LWP groups. A gene ontology analysis revealed that phospholipid, lipid metabolic, apoptotic, lipid biosynthetic, and lipid and fatty acid transport processes were significantly enriched. Potential functional lncRNAs that regulate lipid metabolism, amino acid synthesis, as well as the Janus kinase (JAK)-signal transducer and activator of transcription (STAT) and hedgehog signalling pathways, were identified. Consequently, five lncRNAs (LNC_002171, LNC_000797, LNC_005567, LNC_013595, and LNC_020367) were considered to be potential regulators of hair follicle density and development. Three DE lncRNAs and genes were validated by quantitative real-time polymerase chain reaction (q-PCR).

**Conclusions:**

LncRNA profiles provide information on lncRNA expression to improve the understanding of molecular mechanisms involved in the regulation of hair follicle density.

**Supplementary Information:**

The online version contains supplementary material available at 10.1186/s12864-021-07398-4.

## Background

The Angora rabbit is an economically important livestock breed in several countries, especially in China and France. Wool production is one of the most important traits in Angora rabbits. The fur quality of rabbits is largely dependent on hair density, and hair follicle density determines hair density [1, 2]. For Angora rabbits under the same environmental conditions, gender, body site, and the month of age are closely related to wool fibre production [3]. Genetic factors that influence wool fibre production are fibre diameter, length, fineness, and the fibre density [3–7]. The mean hair follicle density depends on the skin area. The development of wool follicles occurs during prenatal life and no new hair follicles are formed after birth, implying that hair density in an adult rabbit will depend on how much that particular body part grows after the formation of the hair follicles [7, 8]. Correspondingly, hair follicle density and other wool characteristics are highly variable over the human and rabbit body [7–9]. The molecular mechanism underlying hair follicle density in rabbit skin and hair follicle development remains unclear.

Hair follicle development is a complex morphogenetic process and undergoes periodic stages of growth (anagen), regression (catagen), and relative quiescence (telogen) [10–12]. The process of hair follicle formation and differentiation relies on many regulating molecules including messenger RNAs (mRNAs) and micro RNAs (miRNAs) [13–15], as well as a variety of signalling systems, such as the Wnt, Notch, bone morphogenetic protein (BMP), and fibroblast growth factor (FGF) pathways [16–21]. LncRNAs are RNA transcripts longer than 200 nucleotides that lack open reading frames (ORF) and protein-coding capabilities [22]. They regulate protein-coding gene expression at posttranscriptional and transcriptional levels [23, 24]. It is generally known that lncRNAs are also involved in the regulation of the hair follicle development and skin homeostasis [25–27]. RP11-766 N7.3, H19 and HOTAIR are specific lncRNAs that are involved in Wnt signalling to regulate hair follicle development [28]. Strand-specific RNA sequencing (ssRNA-seq) also showed that lncRNAs may be considered as potential candidate markers for further study on the molecular mechanisms of hair follicle initiation [29]. However, hair follicle density-related lncRNAs in rabbits have not been profiled so far.

In this study, the RNA-seq based approach was used to determine lncRNA expression levels in Angora rabbits with high wool production (HWP) and low wool production (LWP) after hair follicle density analysis. The results should provide fundamental resources to reveal the regulatory function of lncRNAs in hair follicle density in rabbits, as well as supply information for understanding human hair disorders such as hypotrichosis.

## Results

### Comparison of hair follicle density in high and low wool production rabbits

**Fig. 1:**
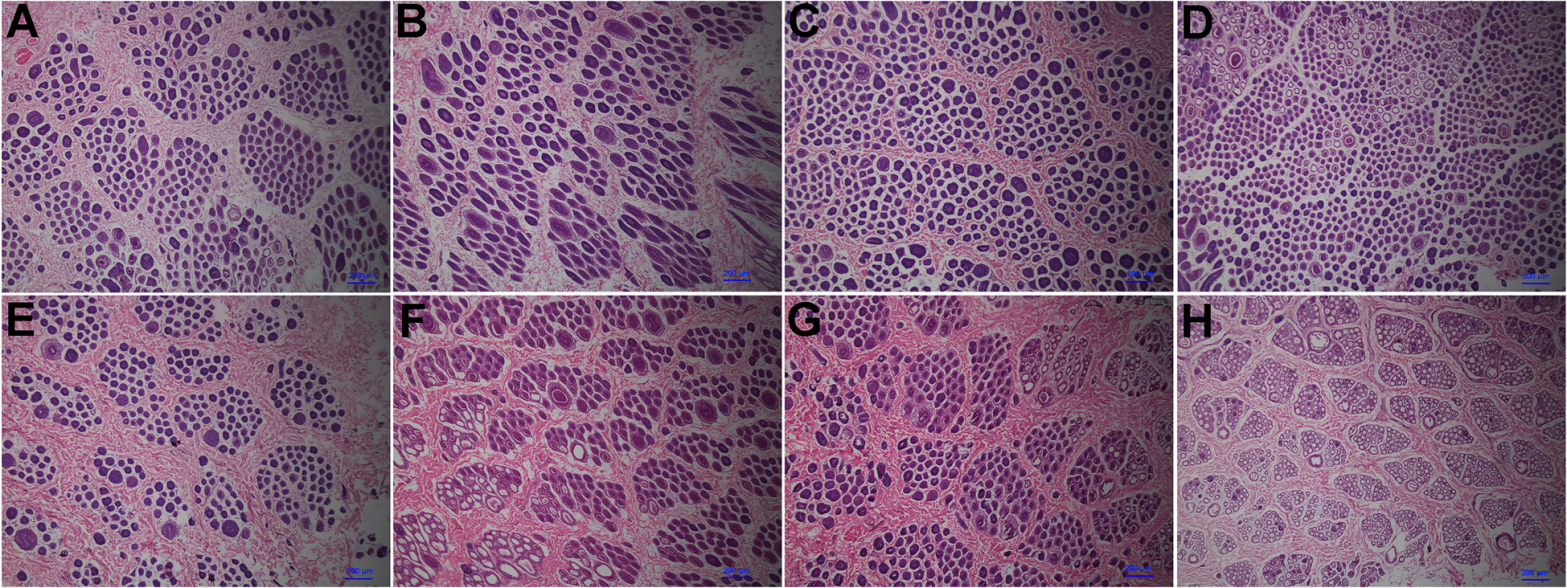
The histological observation of skin tissue from Wan Strain Angora rabbits with HWP and LWP. **a**, **b**, **c**, **d** Transverse section of the backs, abdomens, sides, and hips of Wan Strain Angora rabbits with HWP. **e**, **f**, **g**, **h** Transverse section of the backs, abdomens, sides, and hips of Wan Strain Angora rabbits with LWP. HWP, High wool production; LWP, Low wool production. Bars = 200 μm

To characterize the hair follicle density, the follicle densities of the backs, abdomens, sides, and hips of Wan Strain Angora rabbits with HWP and LWP were compared (Fig. 1). A morphological analysis showed that the hair follicle densities of backs, abdomens, sides, and hips were higher in the HWP group (Fig. 1a, b, c, d) than in the LWP group (Fig. 1e, f, g, h). The results demonstrate that a high hair follicle density leads to high wool production in Wan Strain Angora rabbits.

### Sequencing and assembly

Eight libraries of the HWP groups (H1, H2, H3, and H4) and LWP groups (L1, L2, L3, and L4) were constructed. For the HWP and LWP libraries, above 84,456,770 and 94,769,312 clean reads per sample were obtained, respectively (Table 1). Above 89.19 and 89.02% of the reads were aligned with the rabbit reference genome uniquely located by above 77.55 and 75.67% of the clean reads for the HWP and LWP libraries, respectively. Above 17,380,601 (52.78%) and 21,898,377 (46.66%) reads, respectively, were identified as protein-coding mRNAs of the HWP and LWP groups (Additional file 1: Table S1). The other types of reads amounted to 12,349,910 (36.07%) and 16,461,100 (39.19%) for HWP and LWP groups, respectively, and these reads may include lncRNAs (Additional file 1: Table S1).

**Table 1:** The analyses of reads mapped to the rabbit reference genome

Sample name	H1	H2	H3	H4	L1	L2	L3	L4
Total reads	95,426,664	84,456,770	149,301,000	89,038,716	123,865,064	94,769,312	109,570,388	130,245,954
Total mapped	85,943,449 (90.06%)	75,328,473 (89.19%)	133,972,487 (89.73%)	79,823,704 (89.65%)	110,263,589 (89.02%)	84,819,962 (89.5%)	99,272,110 (90.6%)	117,162,667 (89.95%)
Multiple mapped	11,938,587 (12.51%)	9,315,870 (11.03%)	13,827,931 (9.26%)	10,659,938 (11.97%)	16,534,984 (13.35%)	9,777,660 (10.32%)	14,500,967 (13.23%)	11,066,700 (8.5%)
Uniquely mapped	74,004,862 (77.55%)	66,012,603 (78.16%)	120,144,556 (80.47%)	69,163,766 (77.68%)	93,728,605 (75.67%)	75,042,302 (79.18%)	84,771,143 (77.37%)	106,095,967 (81.46%)
Reads map to ‘+’	36,872,804 (38.64%)	32,897,302 (38.95%)	60,000,375 (40.19%)	34,636,589 (38.9%)	46,454,860 (37.5%)	37,454,686 (39.52%)	42,357,439 (38.66%)	52,928,464 (40.64%)
Reads map to ‘-’	37,132,058 (38.91%)	33,115,301 (39.21%)	60,144,181 (40.28%)	34,527,177 (38.78%)	47,273,745 (38.17%)	37,587,616 (39.66%)	42,413,704 (38.71%)	53,167,503 (40.82%)
Non-splice reads	56,778,516 (59.5%)	51,943,073 (61.5%)	89,625,539 (60.03%)	51,395,189 (57.72%)	74,099,166 (59.82%)	57,787,882 (60.98%)	65,280,836 (59.58%)	79,655,006 (61.16%)
Splice reads	17,226,346 (18.05%)	14,069,530 (16.66%)	30,519,017 (20.44%)	17,768,577 (19.96%)	19,629,439 (15.85%)	17,254,420 (18.21%)	19,490,307 (17.79%)	26,440,961 (20.3%)
Reads mapped in proper pairs	69,994,050 (73.35%)	62,138,530 (73.57%)	113,304,356 (75.89%)	65,315,796 (73.36%)	88,424,106 (71.39%)	70,592,926 (74.49%)	80,480,580 (73.45%)	100,326,142 (77.03%)

### Characterization of lncRNAs in rabbit skin tissue

**Fig. 2:**
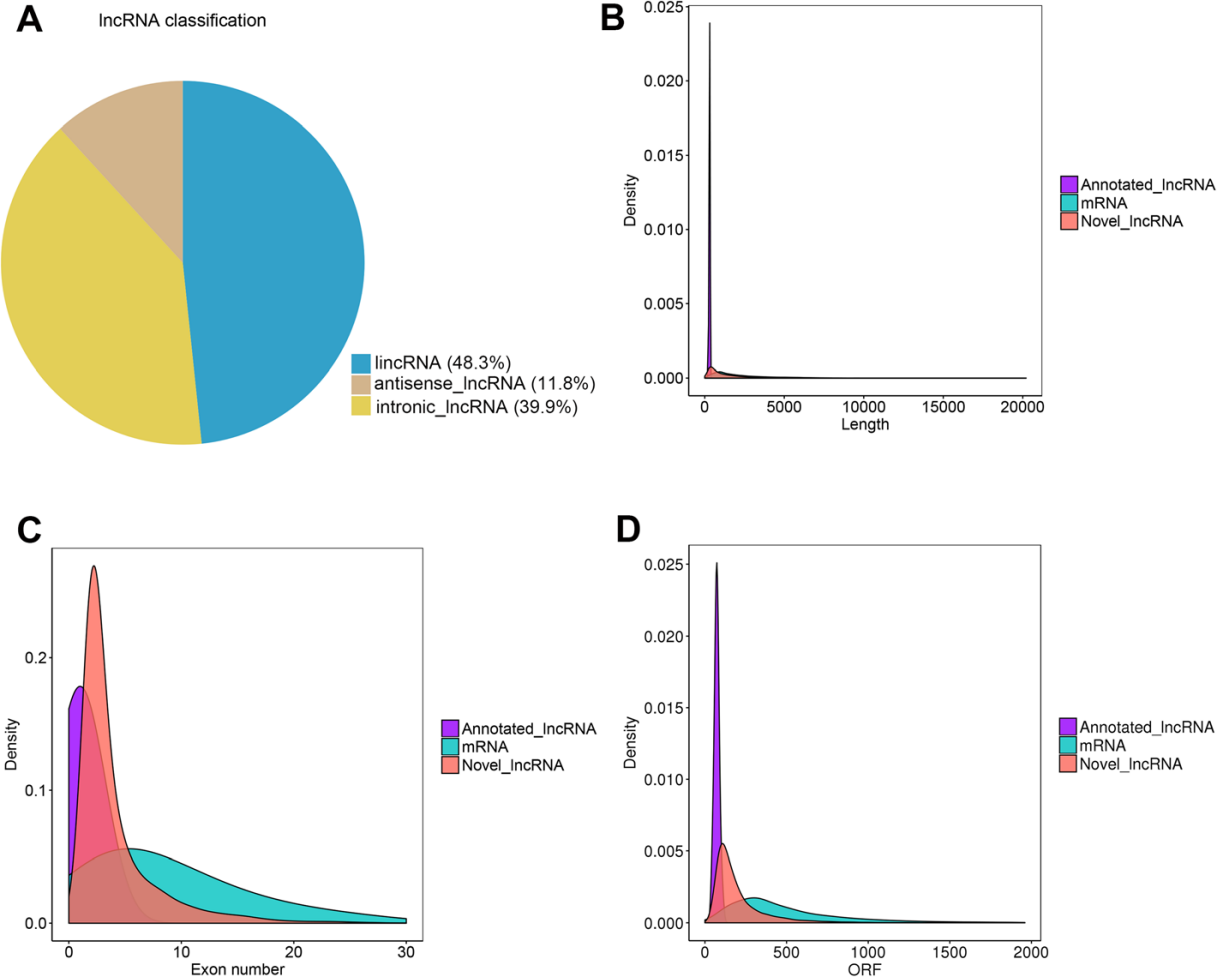
Characterization of lncRNAs transcribed from Wan Strain Angora rabbits. **a** The lncRNA classification in Wan Strain Angora rabbits. **b** Length distribution of lncRNAs and protein-coding transcripts. **c** Exon number distribution per the transcript of lncRNAs and protein-coding transcripts. **d** ORF number distribution per the transcript of lncRNAs and protein-coding transcripts. ORF, open reading frames

The RNA-seq analysis produced 22,136 lncRNAs (Additional file 2: Table S2). The lncRNA transcripts included 10,692 lincRNAs (48.3%), 2612 antisense lncRNAs (11.8%), and 8832 intronic lncRNAs (39.9%) (Fig. 2a). The average length of the novel lncRNAs was considerably shorter than the mRNAs, but longer than the known lncRNAs (Fig. 2b). The exon numbers of the novel lncRNAs were less than the mRNAs while greater than the known lncRNAs (Fig. 2c). In addition, ORF size in novel lncRNAs was longer than that in annotated lncRNAs, but shorter than that in protein-coding genes (Fig. 2d).

### Long noncoding RNAs and mRNAs expression profiles in rabbit skin tissue

**Fig. 3:**
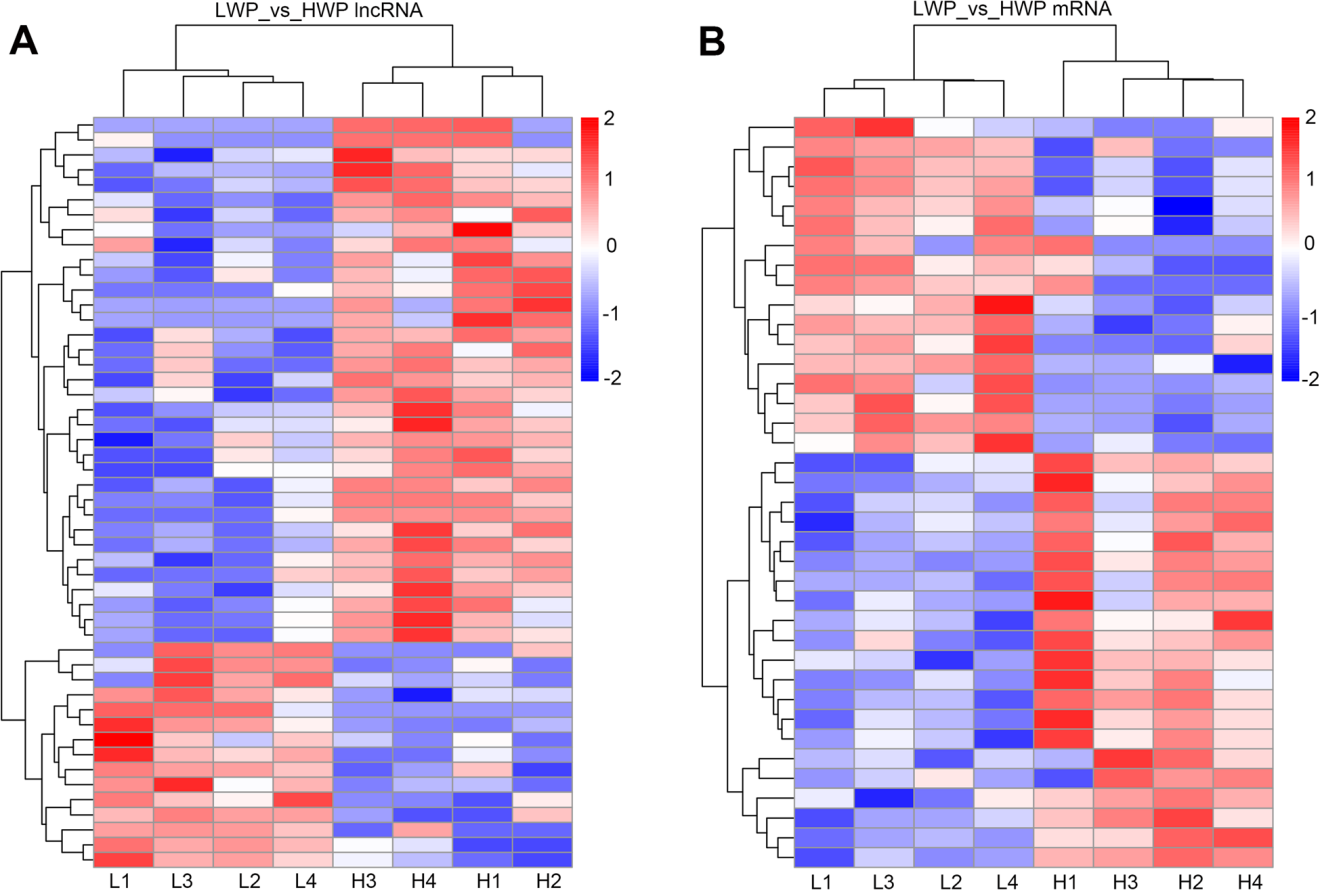
Heatmaps of differentially expressed lncRNAs and mRNAs between HWP and LWP rabbits. **a** lncRNAs. **b** mRNAs. “L” and “H” represent low wool production and high wool production groups, respectively. “Red” and “blue” indicate up-regulated and down-regulated transcripts, respectively. HWP, High wool production; LWP, Low wool production

The results showed that the expression levels of mRNAs were higher than those of lncRNAs (Additional file 3: Fig. S1). Fifty and thirty-eight differentially expressed (DE) lncRNAs and genes, respectively, were screened in the LWP and HWP groups (Additional file 4: Table S3, Table S4). Of these lncRNAs and genes, 15 lncRNAs and 21 genes were upregulated, and 35 lncRNAs and 17 genes were downregulated in the LWP group. Hierarchical cluster analysis of lncRNA and mRNA expression levels between LWP and HWP groups revealed distinct expression patterns (Fig. 3).

### Long noncoding RNA target prediction and functional analysis

**Fig. 4:**
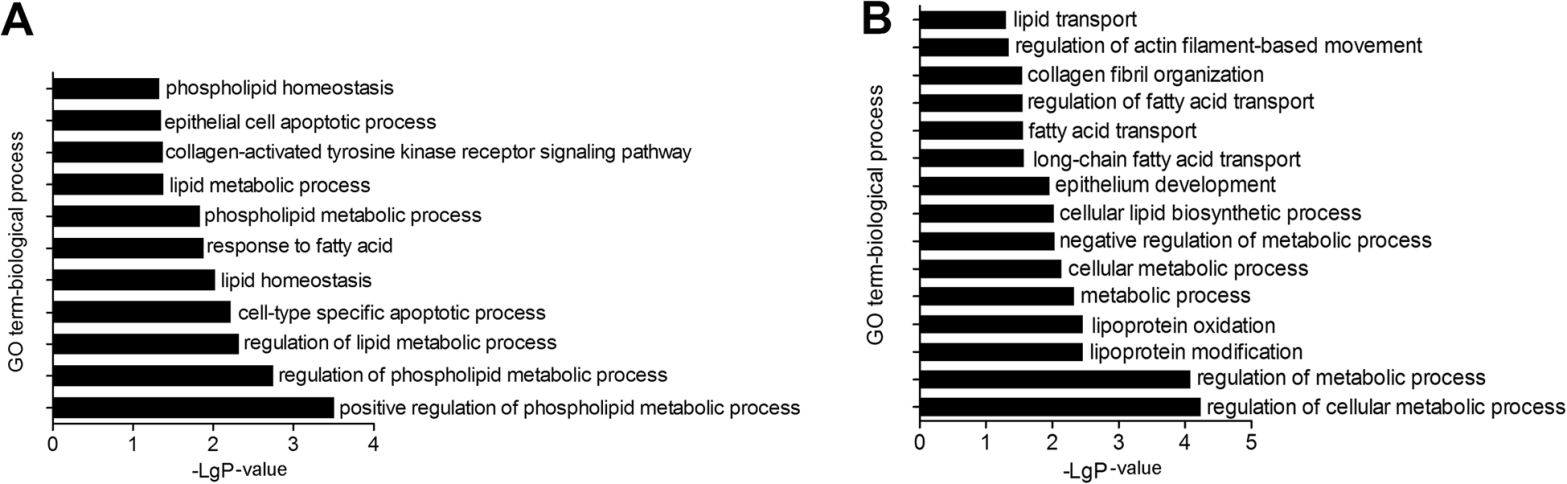
GO enrichment analysis of *cis*-regulated target genes. **a** GO analysis of lncRNA co-location mRNAs according to biological process **b** GO analysis of lncRNA co-expression mRNAs according to biological process. The hierarchical category of the GO terms is biological process. The significance of enrichment of each GO term was assessed by *P*-value < 0.05, and GO terms were subsequently filtered by the enrichment scores (−Lg*P*-value)

**Fig. 5:**
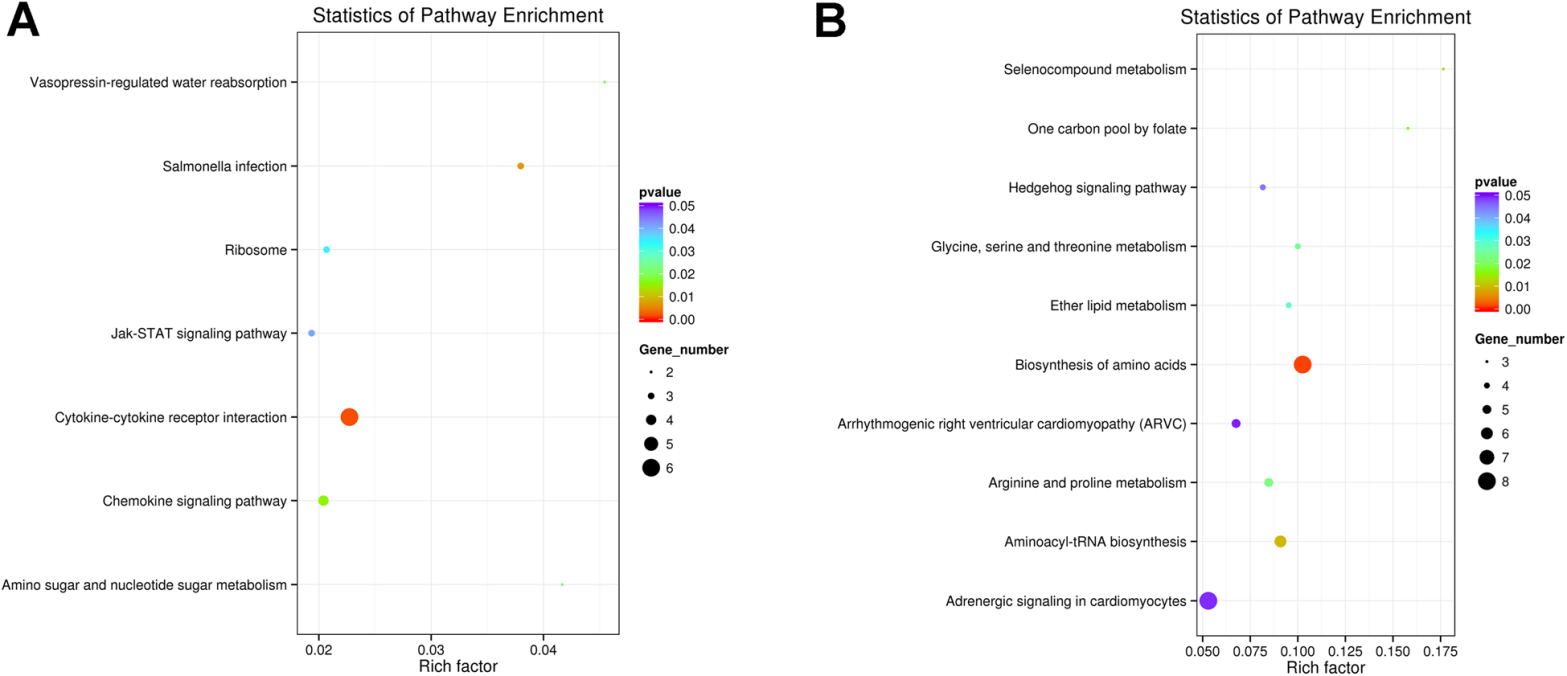
KEGG pathway enrichment analysis of the *cis*-regulated target genes. **a** Pathway enrichment for lncRNA co-location mRNAs. **b** Pathway enrichment for lncRNA co-expression mRNAs. The dot plots present the enrichment of these mRNAs in every pathway. The colour of each dot corresponds to the *P*-value which indicates the significant level of change of each pathway. The size of each dot shows the number of mRNAs involved in the corresponding pathway. The horizontal axis represents the enrichment level of the pathways

**Fig. 6:**
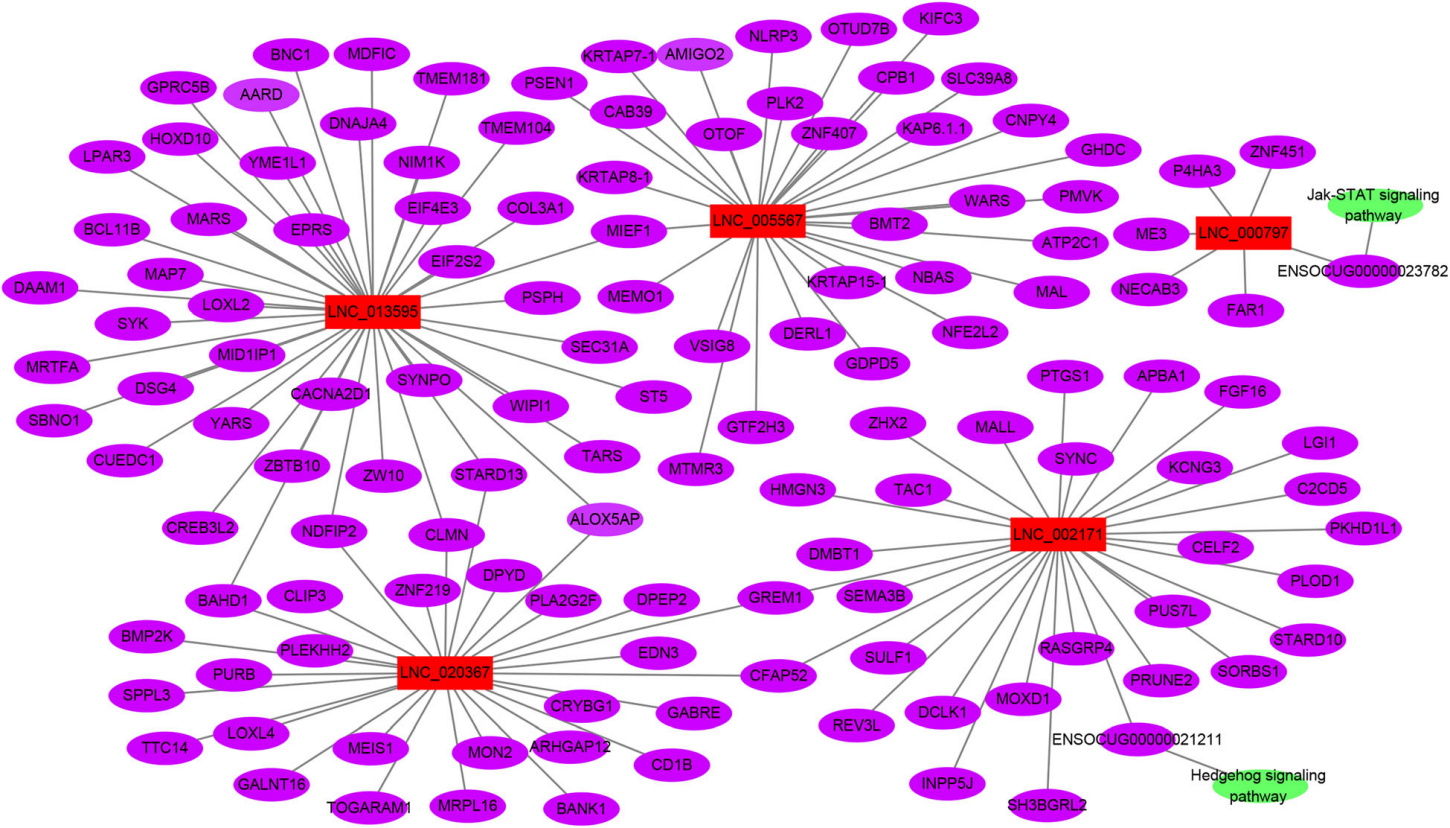
Regulatory networks between lncRNA and mRNA. The purple ellipse represents mRNAs targeted by lncRNAs, the rectangle represents lncRNAs, and the green ellipse represents pathways enriched by mRNAs

The potential target genes of lncRNAs were predicted accordingly their position (co-location) and expression correlation (co-expression) with the protein-coding genes. Gene ontology (GO) analysis was applied to investigate the potential functions of the lncRNAs’ co-location and co-expression mRNAs on the regulation of hair follicle development and wool production (Fig. 4). The significance of enrichment of each GO term was assessed by *P*-value < 0.05, and then the GO terms were filtered by the enrichment scores (−Lg *P*-value). The GO enrichment analysis showed that the lncRNAs’ co-location mRNAs were significantly enriched in phospholipid, lipid metabolic, and epithelial cell apoptotic processes in the biological process category (Fig. 4a), while co-expression mRNAs were significantly enriched in the cellular metabolic, lipoprotein, lipid biosynthetic, lipid, and fatty acid transport processes (Fig. 4b). The Kyoto Encyclopedia of Genes and Genomes (KEGG) pathway analysis offered a reliable way of elucidating the candidate biological pathways that the integrated target genes were enriching. The cytokine-cytokine receptor interaction, chemokine signalling pathway and JAK-STAT signalling pathway were significantly involved in lncRNAs’ co-location mRNAs (Fig. 5a). In addition, pathways related to the biosynthesis of amino acids, arginine and proline metabolism, ether lipid metabolism, and the hedgehog signalling pathway were highly enriched by lncRNAs’ co-expression mRNAs (Fig. 5b). Therefore, the target genes of the DE lncRNAs between the LWP and HWP groups were related to lipid metabolism, amino acid synthesis, JAK-STAT, and the hedgehog signalling pathway. According to the functional enrichment analyses, five DE lncRNAs (LNC_002171, LNC_000797, LNC_005567, LNC_013595, and LNC_020367) were selected to construct regulatory networks (Fig. 6). LNC_002171 and LNC_000797 were involved in JAK-STAT and the hedgehog signalling pathway.

### Validation of DE lncRNAs and mRNAs with quantitative real-time polymerase chain reaction

**Fig. 7:**
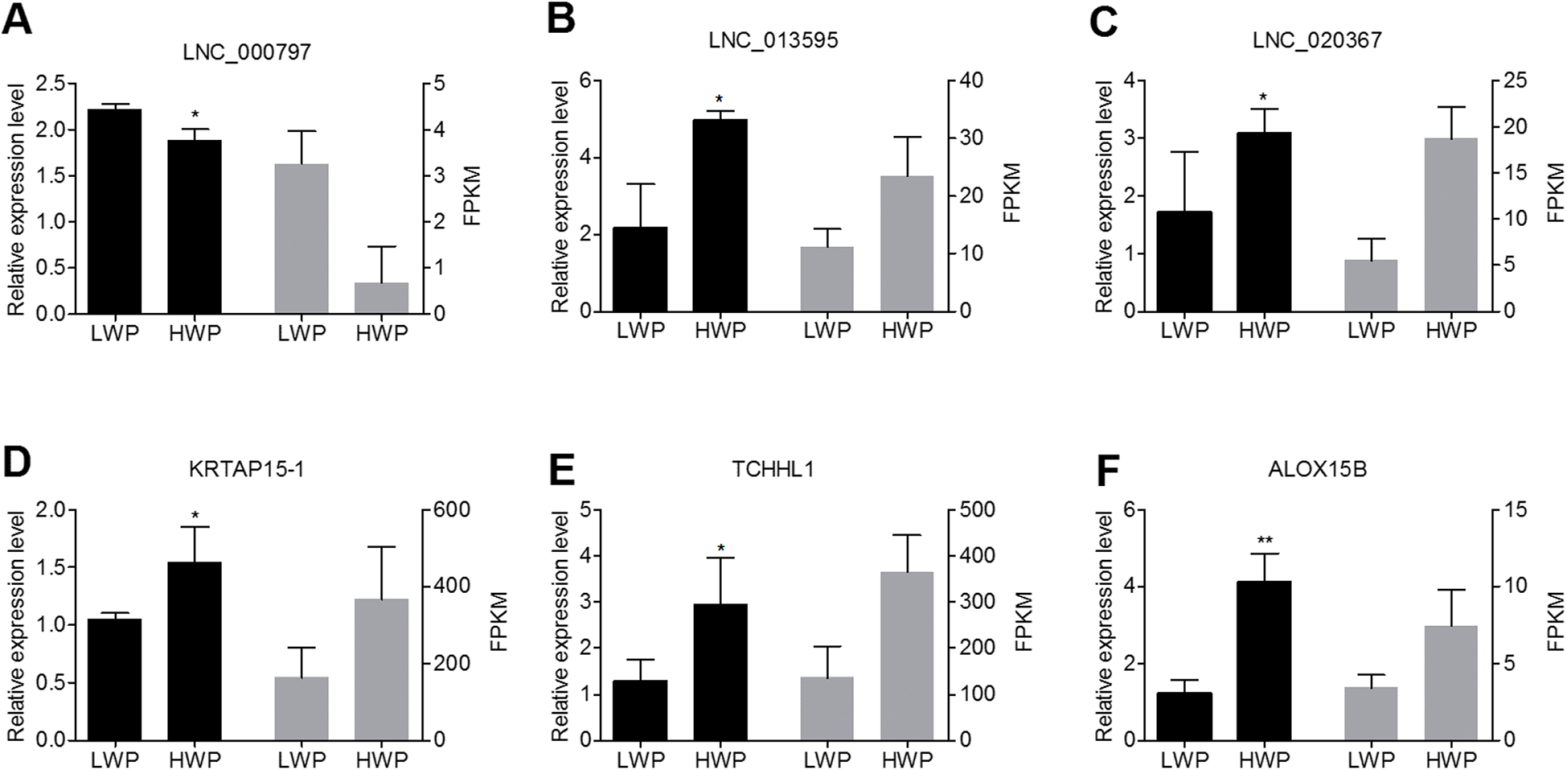
Validation of DE lncRNAs and mRNAs by q-PCR. **a** LNC_000797 **b** LNC_013595 **c** LNC_020367 **d** KRTAP15–1 **e** TCHHL1 **f** ALOX15B. The black and grey columns represent the q-PCR and sequencing results, respectively. LWP represents Wan Strain Angora rabbits with low wool production; HWP represents Wan Strain Angora rabbits with high wool production. FPKM, fragments per kilobase of transcript per million fragments mapped. DE lncRNAs, differentially expressed lncRNAs. q-PCR, quantitative real-time polymerase chain reaction. GAPDH was used as a reference gene to normalize q-PCR data. Bars represent the standard error. ^**^P < 0.01, ^*^P < 0.05

To validate the RNA-Seq results, LNC_000797, LNC_013595, LNC_020367, *KRTAP15–1*, *TCHHL1*, and *ALOX15B* were selected and their expression patterns in the LWP and HWP groups were examined by q-PCR. The results showed that the three DE lncRNAs and mRNAs were differentially expressed in the LWP and HWP groups. In addition, they exhibited a similar trend in the results of the RNA-seq and the q-PCR (Fig. 7). Therefore, the fragments per kilobase of transcript per million mapped reads (FPKM) obtained from RNA-seq could be reliably used to determine lncRNA and mRNA expression in the LWP and HWP groups.

## Discussion

Wool density is one of the most important indices to evaluate the quality of the fur of the Wan Strain Angora rabbit [6]. Hair follicle density determines wool density [1]. The quality of fur is associated mainly with the traits of the hair follicles [30]. To characterize the hair follicle density, the follicle density of the backs, abdomens, sides, and hips of Wan Strain Angora rabbits with HWP and LWP was compared (Fig. 1). A morphological analysis showed that the hair follicle density of backs, abdomens, sides, and hips of the HWP group was higher compared to the LWP group (Fig. 1). The results demonstrated that high hair follicle density contributed to high wool production in the Wan Strain Angora rabbit. In French Angora rabbits, divergent selection of total fleece weight led to a positive difference of 0.55 genetic standard deviation for secondary to primary follicle ratio (S/P), although a low genetic correlation existed between them [31].

The formation of hair follicles is divided into prenatal hair morphogenesis and the postnatal hair cycle [32]. Once established during embryogenesis, hair follicle density is permanently fixed in postnatal life, and the hair follicle location eventually becomes fixed as a result of anchoring in the subcutis [12]. LncRNAs are widely involved in various biological processes, including the hair follicle cycle [33, 34]. The lncRNA and mRNA expression profiles were compared in the dorsal skin of LWP and HWP rabbits, and 50 and 38 DE lncRNAs and genes were obtained, respectively. These lncRNAs and genes might play crucial roles in regulating hair follicle density, and their differential expression might be the reason for differences in hair follicle density and wool production between HWP and LWP rabbits. Liu et al. (2020) analysed the miRNA effect on hair follicle density in the Rex rabbit [35], but lncRNA related to hair density in rabbits has only been done in the present study.

The GO analysis showed that the DE lncRNAs are potential regulators of phospholipid, lipid metabolic, epithelial cell apoptotic, lipid biosynthetic, and lipid and fatty acid transport processes. Keratin-associated proteins (KRTAPs) play a critical role in cross-linking the keratin intermediate filaments to build a hair shaft [36]. *KRTAP7–1*, *KRTAP8–1*, and *KRTAP15–1* were predicted as the targets of LNC_005567 in this study. *KRTAP7–1* is involved in supporting the mechanical strength and shape of hair [36]. *KRTAP15–1* is expressed in secondary follicles in the skin and associated with fibre diameter [37]. *COL3A1* and *LOXL4* were the target genes of LNC_013595 and LNC_020367, respectively. COL3A1 is one of collagens forming different extracellular matrix (ECM) components [38]. The lysyl oxidase like 4 (LOLX4) enzyme is responsible for initiating covalent cross-linking in collagen fibrils and is involved in providing additional mechanical strength to the ECM [39, 40]. The amount of ECM per cell contributes to the volume of the dermal papilla [41]. Hedgehog and JAK-STAT signalling pathway were significantly enriched by target genes *ENSOCUG00000021211* and *ENSOCUG00000023782* of LNC_002171 and LNC_000797, respectively. The hedgehog signalling pathway is correlated to the initiation of hair follicle formation and is a pivotal growth signal for dermal papilla maturation and growth [12, 42, 43]. The JAK-STAT signalling pathway is involved in maintaining the quiescence of hair follicles during telogen [44], and JAK-STAT inhibition contributes to the promotion of hair growth and the activation of hair follicle stem cells [45]. These findings demonstrate that LNC_002171, LNC_000797, LNC_005567, LNC_013595, and LNC_020367 are potentially important regulators of hair follicle density and development.

TCHHL1 is a hair-specific protein given its high expression in scalp and chin skin [46]. TCHHL1 was identified in a genome-wide association study (GWAS) to have a significant association with hair shape within the top-associated single nucleotide polymorphisms (SNPs) (rs17646946), and showed nominally significant association with hair curliness [47]. ALOX15B is restricted to terminally differentiating keratinocytes (in particular the stratum granulosum) and 8(S)-lipoxygenase activity seems to be involved in terminal differentiation of mouse epidermis [48]. Clements et al. (2012) identified reduced expression of *ALOX15B* gene in ankyloblepharon–ectodermal defects–clefting (AEC) syndrome skin, with downregulated genes (*KRT25* and *KRT27*) encoding keratins involved in the morphogenesis of hair follicles [49]. Thus, in combination with the current research, three genes may participate in the regulation of hair follicle density in Angora rabbits. The results of q-PCR of LNC_000797, LNC_013595, LNC_020367, *KRTAP15–1*, *TCHHL1*, and *ALOX15B* showed similar expression patterns between RNA-Seq and q-PCR, demonstrating the reliability of these data.

## Conclusions

In conclusion, differences in the histology and lncRNA profiles of skin were identified in HWP and LWP rabbits. The histological analysis showed a higher hair follicle density in HWP rabbits. The analyses of lncRNA profiles identified candidate lncRNAs involved in lipid metabolism, apoptosis, and hair follicle development. Further studies are required to investigate the roles of candidate lncRNAs in hair follicle density to improve rabbit breeding programmes.

## Methods

### Animals

All animals were procured from the rabbit farm and acquired an approval from the farm owner in the Animal Husbandry and Veterinary Medicine Institute of Anhui Academy of Agriculture Sciences, Hefei, Anhui, China. Sixty Wan Strain Angora rabbits (about 1 year old) were reared in the same conditions with regular pellets and water *ad libtum.* The wool weight of five successive collections using electric shears in 1 year from adult rabbits were determined. The 60 rabbits were divided into two populations designated as high wool production (HWP) and low wool production (LWP) according to wool production. The average wool weights showed remarkable difference (HWP: 401.3 ± 36.5 g vs LWP: 314.4 ± 29.2 g, *P* < 0.001). Finally, four rabbits with high and low wool production (430.1 ± 16.5 g vs 291.6 ± 13.3 g, *P* < 0.0001) were selected for the present study, respectively (Additional file 5: Table S5). The 52 remaining rabbits were reared like ordinary rabbits for wool production.

### Sample collection, preparation for histological examination

The eight rabbits selected (four rabbits with high wool production, four rabbits with low wool production) were anesthetized by injecting 0.7% pentobarbital sodium (6 ml/kg) into ear vein of the rabbits before sampling. Skin tissue samples (1 cm^2^) were collected from the backs, abdomens, sides and hips at the fourth week after plucking for histological analysis. Each skin sample was cut apart into two and then one piece was prepared and then subjected to histological analysis as our previous study [13]. The iodine solution was smeared on the resultant lesion to prevent bacterial infection. After the experiment, the rabbits were retained in the rabbit farm and reared and protected from external stimuli.

### cDNA library construction and sequencing

Under anesthesia, skin samples from the back of the eight rabbits selected (four rabbits with high wool production and four rabbits with low wool production; 430.1 ± 16.5 g vs 291.6 ± 13.3 g, *P* < 0.0001) were collected at the fourth week after plucking for RNA-seq. The skin samples were firstly frozen in liquid nitrogen immediatelly after cutting and then stored at − 80 °C before RNA extration. Whole RNA was extracted from the skin of HWP rabbits (designated as H1, H2, H3, and H4) and LWP rabbits (designated as L1, L2, L3, and L4) using TRIzol reagent (Invitrogen, Carlsbad, CA, USA) according to the manufacturer’s instructions. The samples were sent to the Beijing Novogene Co., LTD with drikold. The purity and integrity of RNA was evaluated using the NanoPhotometer spectrophotometer (Implen, CA, USA) and Agilent 2100 Bioanalyzer (Agilent Technologies, CA, USA). The RNA was purified by removing rRNA, fragmented randomly, and converted to double cDNA, then were ligated with NEBNext adaptors. Finally, eight libraries were created by PCR using the NEBNext® Ultra™ Directional RNA Library Prep Kit (NEB, USA), quantified with Qubit2.0, and sequenced on an Illumina HiSeq 2500 platform (Illumina, San Diego, CA, USA).

### Mapping, assembling and screening

Impurity data were removed from the raw reads and more than 12 Gb clean reads per sample were generated. The clean reads with high quality were then aligned using HISAT2 to the rabbit reference genome (https://www.ncbi.nlm.nih.gov/genome/?term=Rabbit) sequence. The mapped reads of each sample were assembled by StringTie (v2.0.4) [50]. The candidate lncRNAs were distinguished according to its sequence charecteristics (length > 200 nt and noncoding potential) and meantime transcripts predicted with coding potential were filtered out by multiple tools. Conservative and comparative analyses were done between lncRNAs and mRNAs, and classification of lncRNAs was also analyzed.

### Quantification, target prediction and function analysis

The expression levels of the lncRNAs and mRNAs in each sample were calculated by fragments per kilobase of transcript per million fragments mapped (FPKM). Differential expression between LWP and HWP groups was analyzed by using cuffdiff (https://www.genepattern.org/modules/docs/Cuffdiff/7), and the threshold was set as |log2 (Fold Change)| ≥ 1 and *P* value < 0.05. Target prediction was conducted by searching coding genes 100 kb up- and down-stream of lncRNAs (co-location) and analyzing co-expression relationship (pearson correlation) of mRNAs to lncRNAs. Then, GO and KEGG enrichment analyses were performed on targets and the function of key lncRNAs were predicted. GOseq R package [51] and KOBAS (http://www.genome.jp/kegg/) were used to conduct GO and KEGG enrichment analyses.

### Quantitative real-time polymerase chain reaction

Three candidate lncRNAs and mRNAs were selected from the list of DE lncRNAs and DEGs for validation, and the relative expression level was determined by q-PCR on LightCycler 96 (Roche, Switzerland) using TransStart Green qPCR SuperMix (Transgen, Beijing, China) as our previous study [13]. The primers for q-PCR are listed in Additional file 6: Table S6. The reaction was performed in triplicates for each sample (HWP group: H1, H2, H3, and H4; LWP group: L1, L2, L3, and L4). The 2^-ΔΔCT^ method was used to determine the relative expression level of each gene.

### Statistical analyses

Student’s *t*-test with two-sided was used in statistical comparisons in wool weight between HWP and LWP groups and RNA expression. Error bars represent the mean ± standard deviation (SD) as determined using GraphPad Prism 5 (GraphPad Sofware, Inc., La Jolla, CA, USA). A *P* value < 0.05 were considered the criterion for statistical significance.

## Supplementary Information

**Additional file 1: Table S1.** The analyses of reads mapped to the Rabbit reference genome.

**Additional file 2: Table S2.** List of 22,136 annotated lncRNA.

**Additional file 3: Figure S1.** Expression level analysis of the lncRNAs and protein-coding genes.

**Additional file 4: Table S3, Table S4.** Differentially expressed lncRNAs and genes between the LWP and HWP groups, respectively. HWP, High wool production; LWP, Low wool production.

**Additional file 5: Table S5.** The wool production of the rabbits. “L” and “H” represent low wool production and high wool production groups, respectively.

**Additional file 6: Table S6.** Primers for q-PCR. F^1^, forward primer. R^2^, reverse primer.

## Data Availability

The data was presented in the manuscript and the supporting materials. The raw reads data was submitted to the Short Read Archive (SRA) under the accession number SRP299630 and BioProject accession number PRJNA688082 (https://www.ncbi.nlm.nih.gov/sra/PRJNA688082).

## Abbreviations

BMP: Bone morphogenetic protein
DE: Differentially expressed
ECM: Extracellular matrix
FDR: False discovery rate
FGF: Fibroblast growth factor
FPKM: Fragments per kilobase of transcript per million fragments mapped
GO: Gene Ontology
HE: Haematoxylin-eosin
HWP: High wool production
JAK: Janus kinase
KEGG: Kyoto Encyclopedia of Genes and Genomes
lncRNA: long noncoding RNA
LWP: Low wool production
ORF: Open reading frames
q-PCR: Quantitative real-time polymerase chain reaction
SD: Standard deviation
ssRNA-seq: Strand-specific RNA sequencing
STAT: signal transducer and activator of transcription

## References

[1] Paus R, Cotsarelis G (1999). The biology of hair follicles. N Engl J Med.

[2] Sun H, Zhang Y, Bai L, Wang Y, Yang L, Su W (2019). Heat stress decreased hair follicle population in rex rabbits. J Anim Physiol Anim Nutr.

[3] Lanszki J, Thébault RG, Allain D, Szendrõ Z, Eiben C (2001). The effects of melatonin treatment on wool production and hair follicle cycle in angora rabbits. Anim Res.

[4] Rafat SA, Allain D, Rochambeau HD (2009). Genetic description of a divergent selection experiment in angora rabbits with overlapping generations. J Anim Breed Genet..

[5] Rahman SU, Wang X, Yu L (2018). Observations on biotic parameters of angora rabbit breed under controlled conditions in different housing systems. Vet World.

[6] Chen SJ, Liu T, Liu YJ, Dong B, Gu ZL (2011). Identification of single nucleotide polymorphisms in the CCNA2 gene and its association with wool density in Rex rabbits. Genet Mol Res.

[7] Otberg N, Richter H, Schaefer H, Blume-Peytavi U, Sterry W, Lademann J (2004). Variations of hair follicle size and distribution in different body sites. J Invest Dermatol..

[8] Jönsson EH, Bendas J, Weidner K, Wessberg J, Olausson H, Wasling HB (2017). The relation between human hair follicle density and touch perception. Sci Rep.

[9] Zhang Y, Xue JY (2012). Different characteristics of rabbit hair and body parts processing. Chin J Rabbit Farming..

[10] Stenn KS, Paus R (2001). Controls of hair follicle cycling. Physiol Rev.

[11] Tamura Y, Takata K, Eguchi A, Kataoka Y (2018). In vivo monitoring of hair cycle stages via bioluminescence imaging of hair follicle NG2 cells. Sci Rep.

[12] Schneider MR, Ruth SU, Ralf P (2009). The hair follicle as a dynamic miniorgan. Curr Biol.

[13] Ding H, Zhao H, Cheng G, Yang Y, Wang X, Zhao X (2019). Analyses of histological and transcriptome differences in the skin of short-hair and long-hair rabbits. BMC Genomics.

[14] Zhao B, Chen Y, Yan X, Hao Y, Zhu J, Weng Q (2017). Gene expression profiling analysis reveals fur development in rex rabbits (Oryctolagus cuniculus). Genome..

[15] Ding H, Cheng G, Leng J, Yang Y, Zhao X, Wang X (2020). Analysis of histological and microRNA profiles changes in rabbit skin development. Sci Rep.

[16] Higgins CA, Petukhova L, Harel S, Ho YY, Drill E, Shapiro L (2014). FGF5 is a crucial regulator of hair length in humans. Proc Natl Acad Sci U S A.

[17] Kulessa H, Turk G, Hogan BL (2000). Inhibition of bmp signaling affects growth and differentiation in the anagen hair follicle. EMBO J.

[18] Demehri S, Kopan R (2009). Notch signaling in bulge stem cells is not required for selection of hair follicle fate. Development..

[19] Hardy MH (1992). The secret life of the hair follicle. Trends Genet.

[20] Oro AE, Scott MP (1998). Splitting hairs. Dissecting roles of signaling systems in epidermal development. Cell..

[21] Lin CM, Yuan YP, Chen XC, Li HH, Cai BZ, Liu Y (2015). Expression of Wnt/beta-catenin signaling, stem-cell markers and proliferating cell markers in rat whisker hair follicles. J Mol Histol.

[22] Lou X, Ma X, Wang D, Li X, Sun B, Zhang T (2019). Systematic analysis of long non-coding RNA and mRNA expression changes in ApoE-deficient mice during atherosclerosis. Mol Cell Biochem..

[23] Biao Y, Zhen-Hua W, Jin-Tao G (2012). The research strategies for probing the function of long noncoding RNAs. Genomics..

[24] Kornienko AE, Guenzl PM, Barlow DP, Pauler FM (2013). Gene regulation by the act of long non-coding RNA transcription. BMC Biol.

[25] Wang S, Ge W, Luo Z, Guo Y, Jiao B, Qu L (2017). Integrated analysis of coding genes and non-coding RNAs during hair follicle cycle of cashmere goat ( Capra hircus ). BMC Genomics.

[26] Song S, Yang M, Li Y, Rouzi M, Zhao Q, Pu Y (2018). Genome-wide discovery of lincRNAs with spatiotemporal expression patterns in the skin of goat during the cashmere growth cycle. BMC Genomics.

[27] Zhu YB, Wang ZY, Yin RH, Jiao Q, Zhao SJ, Cong YY (2018). A lncRNA-H19 transcript from secondary hair follicle of Liaoning cashmere goat: identification, regulatory network and expression regulated potentially by its promoter methylation. Gene..

[28] Chang-Min L, Yang L, Keng H, Xian-Cai C, Bo-Zhi C, Hai-Hong L (2014). Long noncoding RNA expression in dermal papilla cells contributes to hairy gene regulation. Biochem Biophys Res Commun.

[29] Yue Y, Guo T, Yuan C, Liu J, Guo J, Feng R (2016). Integrated analysis of the roles of long noncoding RNA and coding RNA expression in sheep (Ovis aries) skin during initiation of secondary hair follicle. PLoS One.

[30] Chen S, Liu T, Liu Y, Dong B, Gu Z (2011). Gene expression patterns in different wool densities of Rex rabbit using cDNA microarray. Agr Sci China.

[31] Rafat SA, Rochambeau HD, Thébault RG, David I, Deretz S, Bonnet M, et al. Divergent selection for total fleece weight in angora rabbits: correlated responses in wool characteristics. Livest Sci 2008;113(1):0–13.

[32] Choi BY (2018). Hair-growth potential of ginseng and its major metabolites: a review on its molecular mechanisms. Int J Mol Sci..

[33] Lin CM, Liu Y, Huang K, Chen XC, Cai BZ, Li HH (2014). Long noncoding RNA expression in dermal papilla cells contributes to hairy gene regulation. Biochem Bioph Res Co.

[34] Zhao B, Chen Y, Hu S, Yang N, Wang M, Liu M (2019). Systematic analysis of non-coding RNAs involved in the angora rabbit (Oryctolagus cuniculus) hair follicle cycle by RNA sequencing. Front Genet.

[35] Liu G, Li S, Liu H, Zhu Y, Bai L, Sun H (2020). The functions of ocu-miR-205 in regulating hair follicle development in Rex rabbits. BMC Dev Biol.

[36] Arlud S, He N, Sari EM, Ma ZJ, Zhang H, An TW (2017). Highly conserved keratin-associated protein 7-1 gene in yak, taurine and zebu cattle. Folia Biol.

[37] Zhao M, Zhou H, Hickford JGH, Gong H, Wang J, Hu J (2019). Variation in the caprine keratin-associated protein 15-1 (KAP15-1) gene affects cashmere fibre diameter. Arch Anim Breed.

[38] Li B, Qiao L, An L, Wang W, Liu J, Ren Y (2018). Transcriptome analysis of adipose tissues from two fat-tailed sheep breeds reveals key genes involved in fat deposition. BMC Genomics.

[39] Clarke DL, Carruthers AM, Mustelin T, Murray LA (2013). Matrix regulation of idiopathic pulmonary fibrosis: the role of enzymes. Fibrogenesis Tissue Repair.

[40] Leask A (2015). Matrix remodeling in systemic sclerosis. Semin Immunopathol.

[41] Elliott K, Stephenson TJ, Messenger AG (2000). Differences in hair follicle dermal papilla volume are due to extracellular matrix volume and cell number: implications for the control of hair follicle size and androgen responses. J Invest Dermatol.

[42] Chiang C, Swan RZ, Grachtchouk M, Bolinger M, Litingtung Y, Robertson EK (1999). Essential role for sonic hedgehog during hair follicle morphogenesis. Dev Biol.

[43] St-Jacques B, Dassule HR, Karavanova I, Botchkarev VA, Li J, Danielian PS (1998). Sonic hedgehog signaling is essential for hair development. Curr Biol.

[44] Wang E, Harel S, Christiano AM (2016). JAK-STAT signaling jump starts the hair cycle. J Invest Dermatol..

[45] Harel S, Higgins CA, Cerise JE, Dai Z, Chen JC, Clynes R (2015). Pharmacologic inhibition of JAK-STAT signaling promotes hair growth. Sci Adv.

[46] Liu F, Chen Y, Zhu G, Hysi PG, Wu S, Adhikari K (2018). Meta-analysis of genome-wide association studies identifies 8 novel loci involved in shape variation of human head hair. Hum Mol Genet.

[47] Wu Z, Latendorf T, Meyer-Hoffert U, Schroder JM (2011). Identification of trichohyalin-like 1, an s100 fused-type protein selectively expressed in hair follicles. J Invest Dermatol..

[48] Furstenberger G, Marks F, Krieg P (2002). Arachidonate 8(S)-lipoxygenase. Prostaglandins Other Lipid Mediat.

[49] Clements SE, Techanukul T, Lai-Cheong JE, Mee JB, South AP, Pourreyron C (2012). Mutations in AEC syndrome skin reveal a role for p63 in basement membrane adhesion, skin barrier integrity and hair follicle biology. Brit J Dermatol.

[50] Pertea M, Kim D, Pertea GM, Leek JT, Salzberg SL (2016). Transcript-level expression analysis of RNA-seq experiments with HISAT. StringTie and Ballgown Nat Protoc.

[51] Young MD, Wakefield MJ, Smyth GK, Oshlack A (2010). Gene ontology analysis for RNA-seq: accounting for selection bias. Genome Biol.

