# Supplementary material for: Analysis of histology and long noncoding RNAs involved in the rabbit hair follicle density using RNA sequencing
Source: BMC Genomics. 2021 Jan 28;22:89. doi: 10.1186/s12864-021-07398-4 (PMC7845105; doi:10.1186/s12864-021-07398-4)
Supplement: Supplementary file 1 — Additional file 1: Table S1. The analyses of reads mapped to the Rabbit reference genome. [file 12864_2021_7398_MOESM1_ESM.pdf]

Table S1

Sample name	H1	H2	H3	H4	L1	L2	L3	L4
Mt_rRNA	89969 (0.24%)	83737 (0.25%)	123732 (0.21%)	69926 (0.20%)	165703 (0.35%)	74705 (0.20%)	120141 (0.29%)	110151 (0.21%)
Mt_tRNA	1106 (0.00%)	1053 (0.00%)	1658 (0.00%)	1044 (0.00%)	1105 (0.00%)	1236 (0.00%)	1293 (0.00%)	1525 (0.00%)
miRNA	712223 (1.94%)	448107 (1.36%)	977520 (1.64%)	620374 (1.81%)	551478 (1.18%)	607368 (1.62%)	776288 (1.85%)	719617 (1.37%)
misc_RNA	1935579 (5.27%)	1421694 (4.32%)	2923012 (4.90%)	1976916 (5.77%)	1586504 (3.38%)	1990731 (5.32%)	2425073 (5.77%)	2226282 (4.23%)
processed_pseudogene	11623 (0.03%)	8135 (0.02%)	15002 (0.03%)	10862 (0.03%)	10739 (0.02%)	11710 (0.03%)	8804 (0.02%)	16152 (0.03%)
protein_coding	19988707 (54.41%)	17380601 (52.78%)	32225864 (54.03%)	19104245 (55.80%)	21898377 (46.66%)	19949272 (53.29%)	22097640 (52.62%)	28000150 (53.22%)
pseudogene	57976 (0.16%)	45782 (0.14%)	93306 (0.16%)	58613 (0.17%)	51988 (0.11%)	55890 (0.15%)	57386 (0.14%)	79773 (0.15%)
rRNA	1068 (0.00%)	1625 (0.00%)	16691 (0.03%)	6539 (0.02%)	8755 (0.02%)	2359 (0.01%)	2648 (0.01%)	17547 (0.03%)
snRNA	1772 (0.00%)	1390 (0.00%)	2072 (0.00%)	1557 (0.00%)	1551 (0.00%)	1664 (0.00%)	2181 (0.01%)	1862 (0.00%)
snoRNA	37758 (0.10%)	29449 (0.09%)	51142 (0.09%)	39036 (0.11%)	31187 (0.07%)	35716 (0.10%)	46008 (0.11%)	44765 (0.09%)
Others	13900666 (37.84%)	13511566 (41.03%)	23213628 (38.92%)	12349910 (36.07%)	22626181 (48.21%)	14706890 (39.28%)	16461100 (39.19%)	21397818 (40.67%)
