# Supplementary figures and images for: Analysis of histology and long noncoding RNAs involved in the rabbit hair follicle density using RNA sequencing
Source: BMC Genomics. 2021 Jan 28;22:89. doi: 10.1186/s12864-021-07398-4 (PMC7845105; doi:10.1186/s12864-021-07398-4)

**Figure S1**

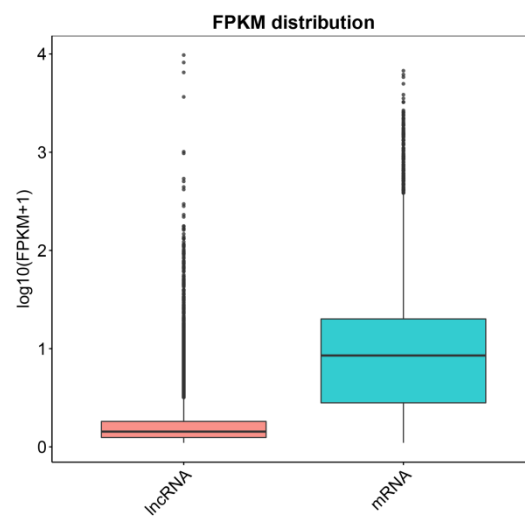

Supplement: Supplementary file 3 — Additional file 3: Figure S1. Expression level analysis of the lncRNAs and protein-coding genes. [file 12864_2021_7398_MOESM3_ESM.pdf]
