# Supplementary material for: Analysis of histology and long noncoding RNAs involved in the rabbit hair follicle density using RNA sequencing
Source: BMC Genomics. 2021 Jan 28;22:89. doi: 10.1186/s12864-021-07398-4 (PMC7845105; doi:10.1186/s12864-021-07398-4)
Supplement: Supplementary file 5 — Additional file 5: Table S5. The wool production of the rabbits. “L” and “H” represent low wool production and high wool production groups, respectively. [file 12864_2021_7398_MOESM5_ESM.pdf]

Table S5

Samples	Wool production (g)	Average wool production (g)
H1	435.5	430.1
H2	449.3	
H3	410.2	
H4	425.4	
L1	305.8	291.6
L2	298.0	
L3	287.3	
L4	275.2	
