# Supplementary material for: Analysis of histology and long noncoding RNAs involved in the rabbit hair follicle density using RNA sequencing
Source: BMC Genomics. 2021 Jan 28;22:89. doi: 10.1186/s12864-021-07398-4 (PMC7845105; doi:10.1186/s12864-021-07398-4)
Supplement: Supplementary file 6 — Additional file 6: Table S6. Primers for q-PCR. F1, forward primer. R2, reverse primer. [file 12864_2021_7398_MOESM6_ESM.pdf]

Table S6

Primer	Sequences of primers (5′-3′)	Size (bp)
LNC_000797-F[1]	TGGTGGCCTGTGACTGAATC	152
LNC_000797-R[2]	CGACAGAGCTGCAAACCCT	
LNC_013595-F	TGTCTCCTCTGCCCTCACAA	144
LNC_013595-R	GCACAGGTGCTCAGATGCTA	
LNC_020367-F	CAGCTGTGGCTCCAGTGCCT	59
LNC_020367-R	ACTGCGAGCAGCAGGGTTT	
KRTAP15-1-F	TAGCCCCTGCCAGACAAATC	117
KRTAP15-1-R	CGGAAGAAGTTGGACCCACA	
TCHHL1-F	CCAAAAACCACCAGCACACG	117
TCHHL1-R	TGGGGCTGAAGACTTCCTCC	
ALOX15B-F	AGGACGCCAGCCATTGAGTA	82
ALOX15B-R	GGTTGAGGCCATTCAGGAAC	
GAPDH-F	AGGTCGGAGTGAACGGATTT	93
GAPDH-R	GATCATTGATGGCGACAACA	
